# Crystal structure and Hirshfeld surface analysis of (*RS*)-3-hy­droxy-2-{[(3a*RS*,6*RS*,7a*RS*)-2-(4-methyl­phenyl­sulfon­yl)-2,3,3a,6,7,7a-hexa­hydro-3a,6-ep­oxy-1*H*-isoindol-6-yl]meth­yl}isoindolin-1-one

**DOI:** 10.1107/S2056989021001626

**Published:** 2021-02-16

**Authors:** Dmitriy F. Mertsalov, Maryana A. Nadirova, Elena A. Sorokina, Marina A. Vinokurova, Sevim Türktekin Çelikesir, Mehmet Akkurt, Irina A. Kolesnik, Ajaya Bhattarai

**Affiliations:** a Department of Organic Chemistry, Peoples’ Friendship University of Russia, (RUDN University), 6 Miklukho-Maklaya St., 117198, Moscow, Russian Federation; b Department of Physics, Faculty of Sciences, Erciyes University, 38039 Kayseri, Turkey; c Laboratory of Organoelement Compounds, Institute of Physical Organic Chemistry, National Academy of Sciences of Belarus, 13 Surganov St., 220072, Minsk, Belarus; d Department of Chemistry, M.M.A.M.C (Tribhuvan University), Biratnagar, Nepal

**Keywords:** crystal structure, ep­oxy­iso­indole group, tetra­hydro­furan ring, pyrrolidine ring, envelope conformation, boat conformation, Hirshfeld surface analysis, IMDAF reaction

## Abstract

The title compound, C_24_H_24_N_2_O_5_S, crystallizes with two independent mol­ecules (*A* and *B*) in the asymmetric unit. In the central ring systems of both mol­ecules, the tetra­hydro­furan rings adopt envelope conformations, the pyrrolidine rings adopt a twisted-envelope conformation and the six-membered ring is in a boat conformation. In mol­ecules *A* and *B*, the nine-membered groups attached to the central ring system are essentially planar (r.m.s. deviations of 0.002 and 0.003 Å, respectively). They form dihedral angles of 64.97 (9) and 56.06 (10)°, respectively, with the phenyl rings. In the crystal, strong inter­molecular O—H⋯O hydrogen bonds and weak inter­molecular C—H⋯O contacts link the mol­ecules, forming a three-dimensional network. In addition weak π–π stacking inter­actions [centroid-to centroid distance = 3.7124 (13) Å] between the pyrrolidine rings of the nine-membered groups of *A* mol­ecules are observed. Hirshfeld surface analysis and two-dimensional fingerprint plots were used to qu­antify the inter­molecular inter­actions present in the crystal, indicating that the environments of the two mol­ecules are very similar. The most important contributions for the crystal packing are from H⋯H (55.8% for mol­ecule *A* and 53.5% for mol­ecule *B*), O⋯H/H⋯O (24.5% for mol­ecule *A* and 26.3% for mol­ecule *B*) and C⋯H/H⋯C (12.6% for mol­ecule *A* and 15.7% for mol­ecule *B*) inter­actions.

## Abstract 2

The title compound crystallizes with two independent mol­ecules in the asymmetric unit. In the crystal, strong inter­molecular O—H⋯O hydrogen bonds and weak inter­molecular C—H⋯O contacts link the mol­ecules, forming a three-dimensional network. In addition, weak π–π stacking inter­actions are observed.

## Chemical context   

Currently, considerable attention is being paid to the development of atom- and step-economic tools in order to obtain new, practically useful materials. Tandem and domino reactions play an important role in this arsenal, since the isolation of inter­mediates is not required in these processes, as all reaction steps occur spontaneously (Tietze & Beifuss, 1993 ▸).

As an example of using such synthetic tools, we proposed the synthesis of compound **3**, which contains three privileged scaffolds, based on the tandem Hinsberg/IMDAF (intra­molecular Diels–Alder furan; Zubkov *et al.*, 2005 ▸, 2014 ▸) reaction strategy (Demircan *et al.*, 2016 ▸; Nadirova *et al.*, 2020 ▸). Substituted sulfonamides are important because of their broad spectrum of biological activities (Anderson *et al.*, 2012 ▸) while 3-hy­droxy­isoindol-1-ones are well-known nitro­gen-containing heterocyclic compounds with a wide range of physiological activity: agonists of muscarinic M2 receptor modulators, anti­microbial activity *etc*. (Stiefl *et al.*, 2003 ▸; Breytenbach *et al.*, 2000 ▸).

**Figure 1:**
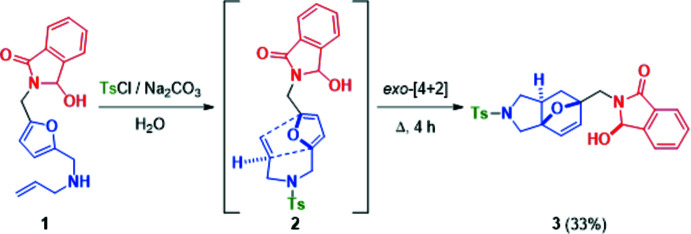
Synthesis of the title compound **3**.

The reaction proceeds smoothly in boiling water. Separation and subsequent crystallization of the resulting solids from ethyl acetate provides the title adduct **3** in moderate yield. The process starts with the Hinsberg *N*-sulfonyl­ation of amine **1**, leading to the formation of the inter­mediate *N*-sulfonamide (**2**), which undergoes spontaneous intra­molecular Diels–Alder reaction. It should be noted that the *exo*-[4 + 2] cyclo­addition proceeds stereoselectively with the exclusive formation of diastereoisomer **3** (Fig. 1 ▸).
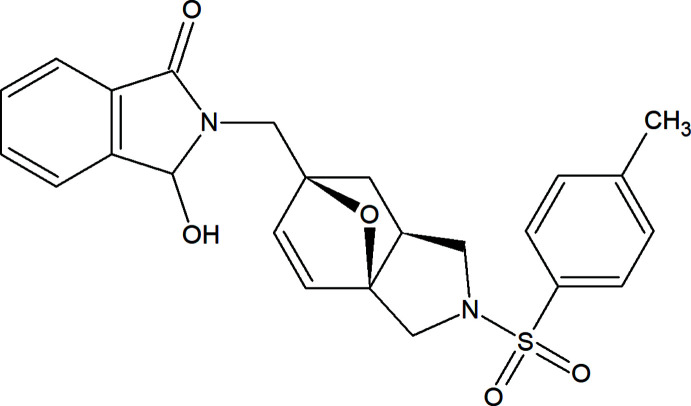


On the other hand, non-covalent inter­actions between mol­ecules play an important role in the synthesis, crystal engineering, mol­ecular recognition, and as key activating/controlling elements in the field of catalysis (Afkhami *et al.*, 2017 ▸; Asadov *et al.*, 2016 ▸; Gurbanov *et al.*, 2017 ▸, 2018 ▸; Karmakar *et al.*, 2017 ▸; Kopylovich *et al.*, 2011*a* ▸,*b* ▸; Ma *et al.*, 2017*a* ▸,*b* ▸; Maharramov *et al.*, 2018 ▸; Mahmoudi *et al.*, 2017 ▸, 2019 ▸; Mahmudov *et al.*, 2010 ▸, 2020 ▸; Mizar *et al.*, 2012 ▸; Sutradhar *et al.*, 2015 ▸). Herein, we highlight the role of weak inter­actions in the structural features of mol­ecule **3**.

## Structural commentary   

**Figure 2:**
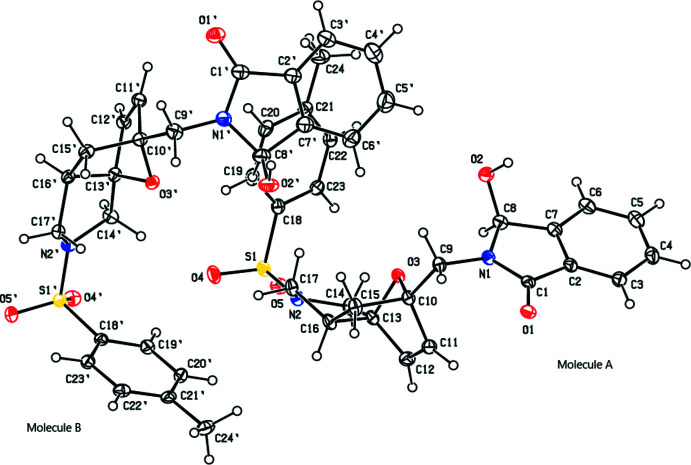
View of the two independent mol­ecules, *A* and *B*, in the asymmetric unit of the title compound **3**, with displacement ellipsoids for the non-hydrogen atoms drawn at the 30% probability level.

As shown Fig. 2 ▸, the title compound **3** crystallizes with two independent mol­ecules (*A* with the atom S and *B* with the atom S′) in the asymmetric unit in which the ep­oxy­iso­indole and phenyl rings are linked through an N—S—C bridge. In the central ring systems of mol­ecules *A* and *B*, the two tetra­hydro­furan rings (*A*: O3/C10–C13, O3/C10/C13/C15/C16 and *B*: O3′/C10′–C13′, O3′/C10′/C13′/C15′/C16′) adopt envelope conformations [puckering parameters (Cremer & Pople, 1975 ▸) *Q* = 0.508 (2), 0.600 (2) and 0.523 (2), 0.602 (2) Å, respectively], the pyrrolidine rings (*A*: N2/C13–C14/C16–C17 and *B*: N2′/C13′–C14′/C16′–C17′) adopt a twisted-envelope conformation [*Q*_T_ = 0.392 (2) Å, φ(2) = 132.8 (4)° and *Q*_T_ = 0.408 (2) Å, φ(2) = 310.0 (3)°, respectively] and the six-membered rings are in a boat conformation (C10–C13/C15/C16; *Q*_T_ = 0.965 (2) Å, θ = 89.90 (12)°, φ = 180.80 (15)° in mol­ecule *A*; C10′–C13′/C15′/C16′, *Q*_T_ = 0.950 (2) Å, θ = 89.90 (12)°, φ = 0.57 (15)° in mol­ecule *B*].

**Figure 3:**
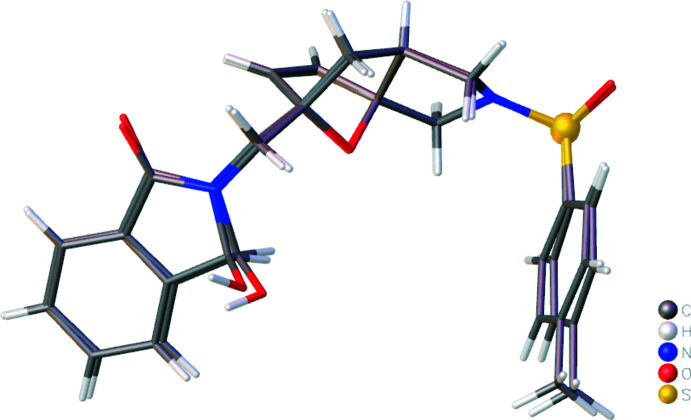
Overlay image of the two mol­ecules (*A* and *B*) in the asymmetric unit of the title compound **3**.

In mol­ecules *A* and *B*, the nine-membered groups (*A*: N1/C1–C8 and *B*: N1′/C1′–C8′) attached to the central ring system are essentially planar (r.m.s deviations of 0.002 and 0.003 Å, respectively). They form dihedral angles of 64.97 (9) and 56.06 (10)°, respectively, with the phenyl rings (*A*: C18–C23 and *B*: C18′–C23′). Fig. 3 ▸ shows the overlay of mol­ecules *A* and *B* in the asymmetric unit (r.m.s. deviation 0.252 Å).

## Supra­molecular features   

**Figure 4:**
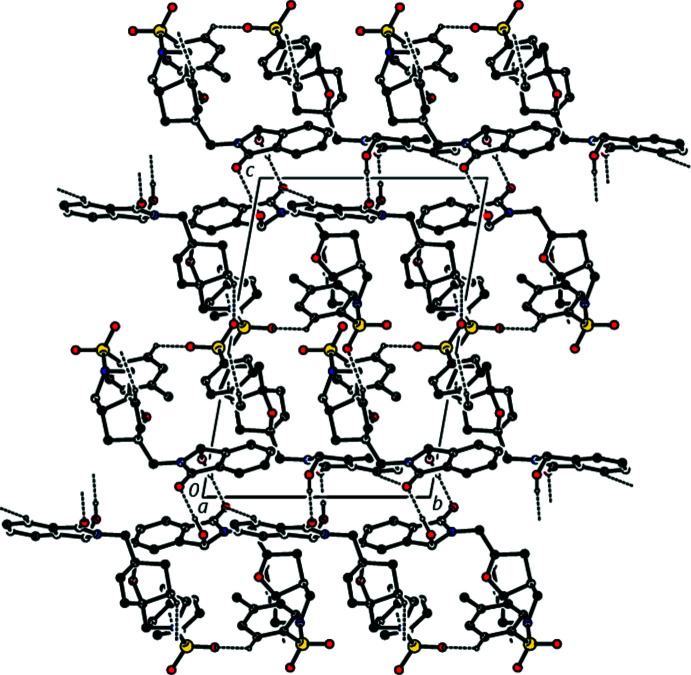
A view of the inter­molecular C—H⋯O and O—H⋯O inter­actions in the crystal structure of the title compound **3**.

In the crystal, strong inter­molecular O—H⋯O hydrogen bonds and weak inter­molecular C—H⋯O contacts link the mol­ecules, forming a three-dimensional network (Table 1 ▸, Fig. 4 ▸). In addition weak π–π stacking inter­actions are observed [*Cg*3⋯*Cg*3(1 − *x*, −*y*, 2 − *z*) = 3.7124 (13) Å where *Cg*3 is the centroid of the pyrrolidine ring (N1/C1/C2/C7/C8) of the nine-membered group in mol­ecule *A*, with slippage of 1.675 Å].

## Hirshfeld surface analysis   

**Figure 5:**
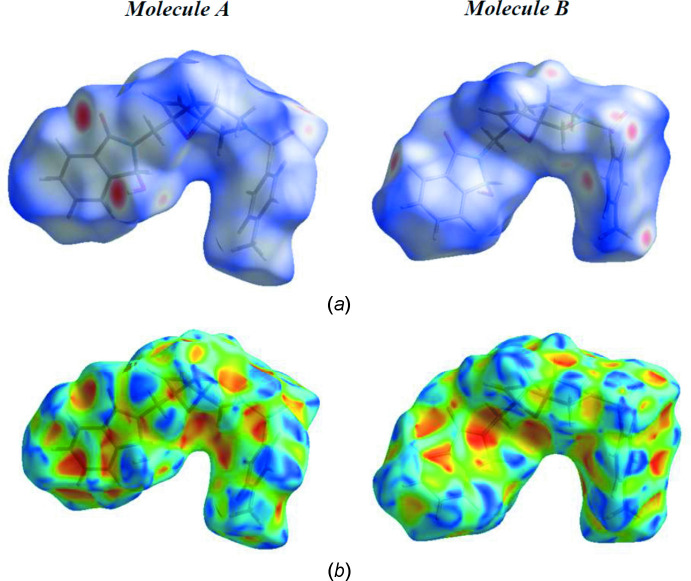
(*a*) View of the three-dimensional Hirshfeld surfaces for mol­ecules *A* and *B* of the title compound **3**; (*b*) Hirshfeld surfaces plotted over shape-index.

The Hirshfeld surfaces for both independent mol­ecules (*A* and *B*) in the asymmetric of the title compound **3** were generated using *Crystal Explorer 17* (Turner *et al.*, 2017 ▸). The *d*_norm_ mappings were performed in the range of −0.6446 to 1.7383 arbitrary units for the mol­ecule *A* and −0.5749 to 1.6904 arbitrary units for mol­ecule *B*. Bold red circles on the *d*_norm_ surfaces (Fig. 5 ▸ *a*) indicate regions of O—H⋯O inter­actions. The C—H⋯O inter­actions also cause red spots on the Hirshfeld surfaces. The shape-index maps (Fig. 5 ▸ *b*) contain red and blue triangles related to π–π inter­actions.

**Figure 6:**
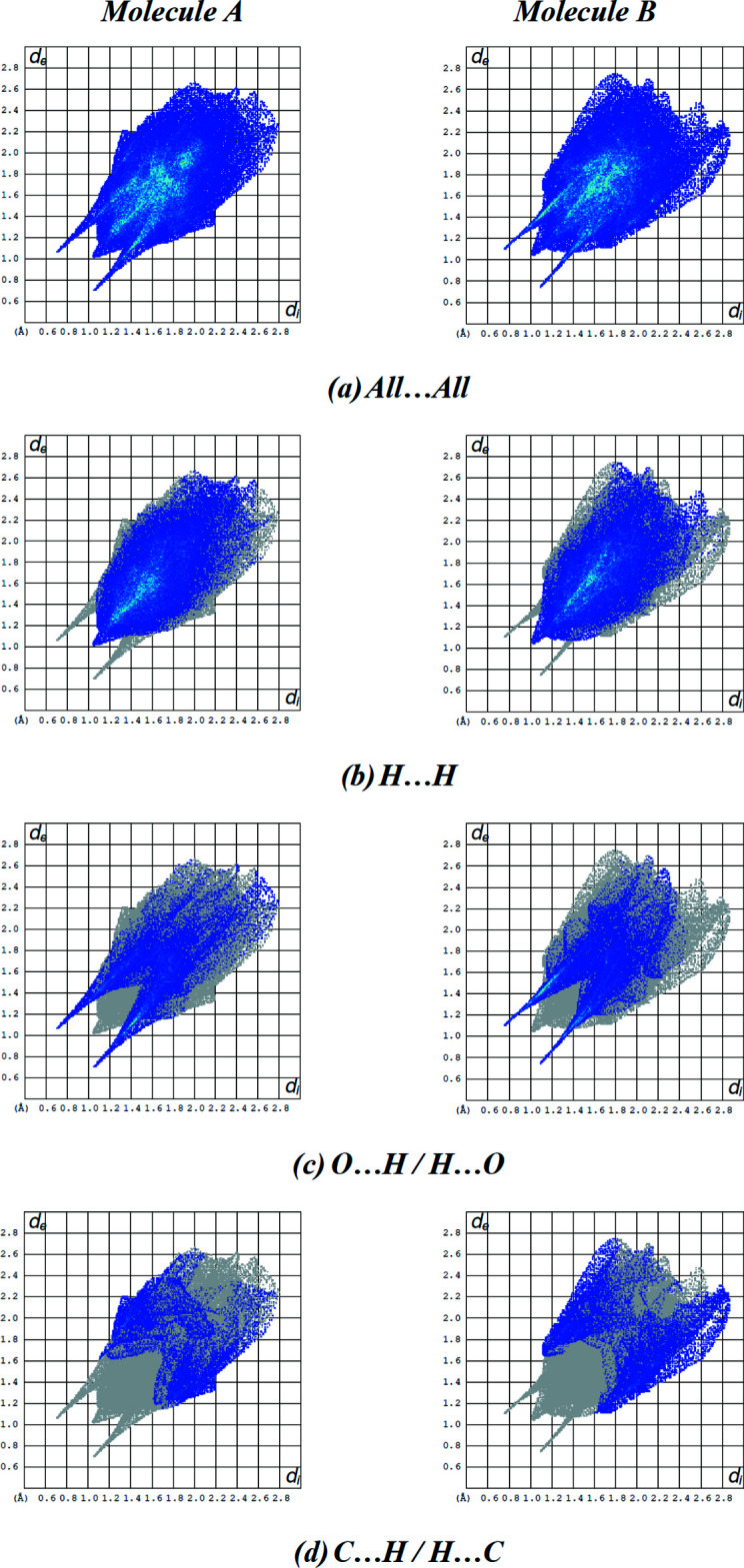
The two-dimensional fingerprint plots for mol­ecules *A* and *B* of the title compound **3** showing (*a*) all inter­actions, and delineated into (*b*) H⋯H, (*c*) O⋯H/H⋯O and (*d*) C⋯H/H⋯C inter­actions. The *d*_i_ and *d*_e_ values are the closest inter­nal and external distances (in Å) from given points on the Hirshfeld surface.

Fingerprint plots (Fig. 6 ▸) reveal that while H⋯H (55.8% for mol­ecule *A* and 53.5% for mol­ecule *B*) inter­actions make the greatest contributions to the surface contacts (Table 2 ▸), as would be expected for a mol­ecule with such a predominance of H atoms, O⋯H/H⋯O (24.5% for mol­ecule *A* and 26.3% for mol­ecule *B*) and C⋯H/H⋯C (12.6% for mol­ecule *A* and 15.7% for mol­ecule *B*) contacts are also substantial. Table 3 ▸ gives the contributions of the other, less significant contacts. As shown in Table 3 ▸, the environments of the two mol­ecules *A* and *B* are very similar. Even the packing looks pseudo-monoclinic, with a pseudo-glide plane relating the two mol­ecules *A* and *B*.

## Database survey   

There are several examples of structures closely related to the 2-(dioxo-λ6-sulfan­yl)octa­hydro-3a,6-ep­oxy­iso­indole skeleton of **3**. Selected examples found in the Cambridge Structural Database (CSD, version 5.40, update of August 2019; Groom *et al.*, 2016 ▸) include (3a*R*,6*S*,7a*R*)-7a-bromo-2-methyl­sulfonyl-1,2,3,6,7,7a-hexa­hydro-3a,6-ep­oxy­iso­indole (CSD refcode ERIVIL; Temel *et al.*, 2011 ▸), (3a*R*,6*S*,7a*R*)-7a-chloro-2-[(4-nitro­phen­yl)sulfon­yl]-1,2,3,6,7,7a-hexa­hydro-3a,6-ep­oxy­iso­indole (AGONUH; Temel *et al.*, 2013 ▸), (3a*R*,6*S*,7a*R*)-7a-chloro-6-methyl-2-[(4-nitro­phen­yl)sulfon­yl]-1,2,3,6,7,7a-hexa­hydro-3a,6-ep­oxy­iso­indole (TIJMIK; Demircan *et al.*, 2013 ▸), (3a*R*,6*S*,7a*R*)-7a-bromo-2-[(4-methyl­phen­yl)sulfon­yl]-1,2,3,6,7,7a-hexa­hydro-3a,6-ep­oxy­iso­indole (UPAQEI; Koşar *et al.*, 2011 ▸), 5-chloro-7-methyl-3-[(4-methyl­phen­yl)sulfon­yl]-10-oxa-3-aza­tri­cyclo­[5.2.1.01,5]dec-8-ene (YAXCIL; Temel *et al.*, 2012 ▸), *tert*-butyl 3a-chloro­perhydro-2,6a-ep­oxy­oxireno(*e*)isoindole-5-carboxyl­ate (MIGTIG; Koşar *et al.*, 2007 ▸) and 2-(2-amino­eth­yl)-3a,4,7,7a-tetra­hydro-1*H*-4,7-ep­oxy­iso­indole-1,3(2*H*)-dione (BILLAL; Mitchell *et al.*, 2013 ▸).

In the crystal of ERIVIL, weak inter­molecular C—H⋯O hydrogen bonds link the mol­ecules into 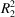
(8) and 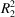
(14) rings along the *b-*axis direction. In the crystal of AGONUH, C—H⋯O hydrogen bonds link the mol­ecules into zigzag chains running along the *b*-axis direction. In the crystal of TIJMIK, two types of C—H⋯O hydrogen bonds generate 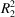
(20) and 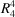
(26) rings, with adjacent rings running parallel to the *ac* plane. Further C—H⋯O hydrogen bonds form a *C*(6) chain, linking the mol­ecules in the *b*-axis direction. In the crystal of UPAQEI, mol­ecules are linked by C—H⋯O hydrogen bonds. In the crystal of YAXCIL, C—H⋯O hydrogen bonds link the mol­ecules into a three-dimensional network. In the crystal of MIGTIG, the mol­ecules are linked only by weak van der Waals inter­actions. The compound BILLAL contains two mol­ecules in the asymmetric unit, which are hydrogen-bonded dimers. The bonds closest to linearity are between the carbonyl groups and the amine H atoms. Inter­molecular hydrogen bonding involving the O atoms also occurs.

## Synthesis and crystallization   

4-Toluene­sulfonyl chloride (0.61 g, 3.2 mmol) was added to 2-({5-[(allyl­amino)­meth­yl]-2-fur­yl}meth­yl)-3-hy­droxy­isoindolin-1-one (0.79 g, 2.7 mmol) in water (10 mL) in the presence of Na_2_CO_3_ (0.34 g, 3.2 mmol). The resulting reaction mixture was refluxed for 4 h and then extracted with DCM (3 × 10 mL). The organic layers were dried with anhydrous MgSO_4_. The desiccator was filtered off, the solution concentrated and the residue was recrystallized from EtOAc. The obtained precip­itate was filtered off, washed with hexane (3 × 5 mL) and dried in air to give 0.4 g (33%) of (*RS*)-3-hy­droxy-2-{[(3a*RS*,6*RS*,7a*RS*)-2-(4-methyl­phenyl­sulfon­yl)-2,3,3a,6,7,7a-hexa­hydro-3a,6-ep­oxy-1*H*-isoindol-6-yl]meth­yl}isoindolin-1-one (**3**) as colourless prisms, m.p. = 468.1–469.1 K. *R*_f_ = 0.6 (EtOH–DMF, 1:2). IR (KBr), ν (cm^−1^): 1167 (ν_s_ SO_2_), 1340 (ν_as_ SO_2_), 1679 (NCO), 3281 (OH). ^1^H NMR (DMSO-*d*_6_, 400 MHz, 301 K): *δ* = 7.74–7.43 (*m*, 8H, HAr), 6.41 (*d*, 1H, OH, *J* = 9.3), 6.37 and 6.22 (2*d*, 2H, H4, H5, *J* = 5.7), 5.68 (*d*, 1H, CH-O, *J* = 9.3), 4.20 (*d*, 1H, NCH_2_ *A*, *J* = 15.3), 3.78 (*d*, 1H, H3*A*, *J* = 12.1), 3.73 (*t*, 1H, H-1*A*, *J* = 9.5), 3.52 (*d*, 1H, NCH_2_ *B*, *J* = 15.3), 3.42 (*d*, 1H, H3*B*, *J* = 12.1), 2.79 (*t*, 1H, H-1*B*, *J* = 9.5), 2.43 (*s*, 3H, CH_3_), 2.00–1.93 (*m*, 1H, H7*A*), 1.55–1.44 (*m*, 2H, H7). ^13^C NMR (DMSO-*d*_6_, 100.4 MHz, 301 K): *δ* = 166.2, 145.0, 143.4, 137.6, 135.5, 133.9, 132.0, 131.1, 129.8, 129.2, 127.2, 123.5, 122.4, 94.7, 92.3, 81.2, 52.8, 48.8, 44.5, 39.7, 33.8, 21.0. MS (APCI): *m*/*z* = 453 [*M* + H]^+^.

## Refinement details   

Crystal data, data collection and structure refinement details are summarized in Table 4 ▸. The hydrogen atoms of the hy­droxy groups were located in a difference-Fourier map and refined freely. The other hydrogen atoms were constrained to ride on their parent atoms with C—H = 0.95, 0.98, 0.99 and 1.00 Å for aromatic, methyl, methyl­ene and methine H atoms, respectively. Isotropic displacement parameters of these atoms were constrained to 1.5*U*_eq_(C) for the methyl and to 1.2*U*_eq_(C) for all other H atoms.

## Supplementary Material

Crystal structure: contains datablock(s) I. DOI: 10.1107/S2056989021001626/zv2004sup1.cif

Structure factors: contains datablock(s) I. DOI: 10.1107/S2056989021001626/zv2004Isup2.hkl

Supporting information file. DOI: 10.1107/S2056989021001626/zv2004Isup3.cml

CCDC reference: 2062492

Additional supporting information:  crystallographic information; 3D view; checkCIF report

## Figures and Tables

**Table 1:** Hydrogen-bond geometry (Å, °)

*D*—H⋯*A*	*D*—H	H⋯*A*	*D*⋯*A*	*D*—H⋯*A*
C12—H12⋯O5^i^	0.95	2.47	3.333 (3)	151
C14—H14*A*⋯O5′^ii^	0.99	2.62	3.506 (3)	149
C23—H23⋯O5′^ii^	0.95	2.41	3.195 (3)	139
C14′—H14*D*⋯O4	0.99	2.58	3.482 (3)	151
C15′—H15*C*⋯O2^iii^	0.99	2.62	3.585 (3)	165
C16′—H16′⋯O4′^iv^	1.00	2.53	3.422 (3)	149
C19′—H19′⋯O4	0.95	2.34	3.078 (3)	134
O2—H2⋯O1^v^	0.92	1.85	2.756 (2)	172
O2′—H2′⋯O1′^vi^	0.90	1.95	2.840 (3)	171

Symmetry codes: (i) -x+1, -y+1, -z+1; (ii) x, y-1, z; (iii) x, y+1, z; (iv) -x+2, -y+2, -z+1; (v) -x+1, -y, -z+2; (vi) -x+2, -y+1, -z+2.

**Table 2:** Summary of short inter­atomic contacts (Å) in the title compound **3**

Contact	Distance	Symmetry operation
O1⋯H3′	2.27	−1 + *x*, *y*, *z*
H2⋯O1	1.85	1 − *x*, −*y*, 2 − *z*
O4⋯H19′	2.34	*x*, *y*, *z*
H8⋯H17*C*	2.41	*x*, −1 + *y*, *z*
H12⋯O5	2.47	1 − *x*, 1 − *y*, 1 − *z*
C20⋯O5	2.411	2 − *x*, 1 − *y*, 1 − *z*
H11⋯H24*B*	2.43	−1 + *x*, *y*, *z*
H15*B*⋯H5	2.42	*x*, 1 + *y*, *z*
H22⋯H14*C*	2.23	2 − *x*, 1 − *y*, 1 − *z*
H2′⋯O1′	1.95	2 − *x*, 1 − *y*, 2 − *z*
H24*F*⋯O4′	2.40	1 − *x*, 2 − *y*, 1 − *z*
H16′⋯O4′	2.53	2 − *x*, 2 − *y*, 1 − *z*
H16′⋯H24*F*	2.41	1 + *x*, *y*, *z*

**Table 3:** Percentage contributions of inter­atomic contacts to the Hirshfeld surfaces for mol­ecules *A* and *B* of the title compound **3**

	Mol­ecule *A*	Mol­ecule *B*
Contact	Percentage contribution	Percentage contribution
H⋯H	55.8	53.5
O⋯H/H⋯O	24.5	26.3
C⋯H/H⋯C	12.6	15.7
C⋯C	3.3	2.6
C⋯O/O⋯C	2.6	0.4
N⋯H/H⋯N	0.8	1.2
C⋯N/N⋯C	0.5	0.1
N⋯O/O⋯N	–	0.1
S⋯H/H⋯S	–	0.1
S⋯H/H⋯S	0.1	–

**Table 4:** Experimental details

Crystal data	
Chemical formula	C_24_H_24_N_2_O_5_S
*M*_r_	452.51
Crystal system, space group	Triclinic, *P*\overline{1}
Temperature (K)	120
*a*, *b*, *c* (Å)	11.8210 (8), 11.8395 (8), 16.7336 (11)
α, β, γ (°)	77.949 (1), 79.555 (1), 77.511 (1)
*V* (Å^3^)	2213.3 (3)
*Z*	4
Radiation type	Mo *K*α
μ (mm^−1^)	0.19
Crystal size (mm)	0.15 × 0.09 × 0.06

Data collection	
Diffractometer	Bruker APEXII CCD
Absorption correction	Multi-scan (*SADABS*; Bruker, 2013 ▸)
*T*_min_, *T*_max_	0.688, 0.746
No. of measured, independent and observed [*I* > 2σ(*I*)] reflections	29692, 13532, 8799
*R*_int_	0.043
(sin θ/λ)_max_ (Å^−1^)	0.717

Refinement	
*R*[*F* ^2^ > 2σ(*F* ^2^)], *wR*(*F* ^2^), *S*	0.060, 0.147, 1.02
No. of reflections	13532
No. of parameters	581
H-atom treatment	H atoms treated by a mixture of independent and constrained refinement
Δρ_max_, Δρ_min_ (e Å^−3^)	0.41, −0.44

Computer programs: *APEX2* and *SAINT* (Bruker, 2013 ▸), *SHELXT* (Sheldrick, 2015*a* ▸), *SHELXL* (Sheldrick, 2015*b* ▸), *ORTEP-3 for Windows* (Farrugia, 2012 ▸) and *PLATON* (Spek, 2020 ▸).

## supplementary crystallographic information

### Crystal data

**Table:**

C_24_H_24_N_2_O_5_S	*Z* = 4
*M**_r_* = 452.51	*F*(000) = 952
Triclinic, *P*1	*D*_x_ = 1.358 Mg m^−^^3^
*a* = 11.8210 (8) Å	Mo *K*α radiation, λ = 0.71073 Å
*b* = 11.8395 (8) Å	Cell parameters from 5394 reflections
*c* = 16.7336 (11) Å	θ = 2.3–27.2°
α = 77.949 (1)°	µ = 0.19 mm^−^^1^
β = 79.555 (1)°	*T* = 120 K
γ = 77.511 (1)°	Prism, colourless
*V* = 2213.3 (3) Å^3^	0.15 × 0.09 × 0.06 mm

### Data collection

**Table:**

Bruker APEXII CCD diffractometer	13532 independent reflections
Radiation source: sealed tube	8799 reflections with *I* > 2σ(*I*)
Graphite monochromator	*R*_int_ = 0.043
φ and ω scans	θ_max_ = 30.6°, θ_min_ = 1.8°
Absorption correction: multi-scan (SADABS; Bruker, 2013)	*h* = −16→16
*T*_min_ = 0.688, *T*_max_ = 0.746	*k* = −16→16
29692 measured reflections	*l* = −23→23

### Refinement

**Table:**

Refinement on *F*^2^	0 restraints
Least-squares matrix: full	Hydrogen site location: mixed
*R*[*F*^2^ > 2σ(*F*^2^)] = 0.060	H atoms treated by a mixture of independent and constrained refinement
*wR*(*F*^2^) = 0.147	*w* = 1/[σ^2^(*F*_o_^2^) + (0.0474*P*)^2^ + 1.6525*P*] where *P* = (*F*_o_^2^ + 2*F*_c_^2^)/3
*S* = 1.02	(Δ/σ)_max_ = 0.001
13532 reflections	Δρ_max_ = 0.41 e Å^−^^3^
581 parameters	Δρ_min_ = −0.44 e Å^−^^3^

### Special details

**Table:**

Geometry. All esds (except the esd in the dihedral angle between two l.s. planes) are estimated using the full covariance matrix. The cell esds are taken into account individually in the estimation of esds in distances, angles and torsion angles; correlations between esds in cell parameters are only used when they are defined by crystal symmetry. An approximate (isotropic) treatment of cell esds is used for estimating esds involving l.s. planes.

### Fractional atomic coordinates and isotropic or equivalent isotropic displacement parameters (Å^2^)

**Table:**

	*x*	*y*	*z*	*U*_iso_*/*U*_eq_	
S1	0.74652 (5)	0.55414 (5)	0.53950 (3)	0.02594 (13)	
O1	0.32628 (13)	0.11057 (14)	0.96738 (9)	0.0263 (3)	
O2	0.71088 (13)	0.03280 (14)	0.88152 (10)	0.0278 (3)	
H2	0.705699	−0.016702	0.931497	0.042 (8)*	
O3	0.57170 (13)	0.31030 (12)	0.74923 (9)	0.0212 (3)	
O4	0.75506 (15)	0.67311 (14)	0.53936 (11)	0.0353 (4)	
O5	0.73338 (14)	0.51910 (15)	0.46520 (10)	0.0317 (4)	
N1	0.50789 (16)	0.12179 (15)	0.89107 (10)	0.0221 (4)	
N2	0.63120 (16)	0.52671 (16)	0.60548 (11)	0.0240 (4)	
C1	0.41649 (19)	0.06714 (19)	0.92534 (12)	0.0216 (4)	
C2	0.44704 (19)	−0.05190 (19)	0.90475 (13)	0.0235 (4)	
C3	0.3823 (2)	−0.1414 (2)	0.92483 (14)	0.0280 (5)	
H3	0.305319	−0.130549	0.954375	0.034*	
C4	0.4354 (2)	−0.2480 (2)	0.89974 (15)	0.0334 (6)	
H4	0.393844	−0.311270	0.912327	0.040*	
C5	0.5482 (2)	−0.2629 (2)	0.85654 (15)	0.0339 (6)	
H5	0.582760	−0.336705	0.840882	0.041*	
C6	0.6117 (2)	−0.1720 (2)	0.83572 (15)	0.0310 (5)	
H6	0.688286	−0.182161	0.805520	0.037*	
C7	0.5589 (2)	−0.06640 (19)	0.86068 (13)	0.0242 (4)	
C8	0.60631 (19)	0.04558 (19)	0.84880 (13)	0.0245 (4)	
H8	0.618437	0.079526	0.788610	0.029*	
C9	0.5108 (2)	0.24198 (19)	0.89628 (13)	0.0237 (4)	
H9A	0.589661	0.245866	0.906416	0.028*	
H9B	0.453931	0.264980	0.943998	0.028*	
C10	0.48228 (19)	0.32883 (18)	0.81931 (13)	0.0217 (4)	
C11	0.3729 (2)	0.3272 (2)	0.78461 (14)	0.0274 (5)	
H11	0.304341	0.300502	0.814352	0.033*	
C12	0.3917 (2)	0.3702 (2)	0.70465 (14)	0.0289 (5)	
H12	0.340100	0.379979	0.665263	0.035*	
C13	0.51173 (19)	0.40013 (19)	0.68953 (13)	0.0230 (4)	
C14	0.5869 (2)	0.4180 (2)	0.60650 (13)	0.0266 (5)	
H14A	0.652259	0.350464	0.601447	0.032*	
H14B	0.540199	0.428583	0.561104	0.032*	
C15	0.4833 (2)	0.45976 (19)	0.82203 (13)	0.0271 (5)	
H15A	0.407907	0.498358	0.849741	0.032*	
H15B	0.547856	0.466503	0.850003	0.032*	
C16	0.5028 (2)	0.51120 (19)	0.72882 (13)	0.0262 (5)	
H16	0.434974	0.573493	0.712567	0.031*	
C17	0.6189 (2)	0.5493 (2)	0.69070 (14)	0.0268 (5)	
H17A	0.615310	0.633446	0.691189	0.032*	
H17B	0.684024	0.501509	0.719696	0.032*	
C18	0.86926 (19)	0.46288 (19)	0.57944 (13)	0.0237 (4)	
C19	0.9263 (2)	0.5017 (2)	0.63118 (15)	0.0295 (5)	
H19	0.901762	0.579077	0.643101	0.035*	
C20	1.0194 (2)	0.4264 (2)	0.66527 (14)	0.0298 (5)	
H20	1.059570	0.453139	0.699867	0.036*	
C21	1.0548 (2)	0.3122 (2)	0.64953 (14)	0.0268 (5)	
C22	0.9972 (2)	0.2756 (2)	0.59696 (14)	0.0257 (5)	
H22	1.021707	0.198304	0.584974	0.031*	
C23	0.90444 (19)	0.3498 (2)	0.56163 (13)	0.0246 (4)	
H23	0.865580	0.323813	0.525833	0.030*	
C24	1.1564 (2)	0.2296 (2)	0.68509 (17)	0.0381 (6)	
H24A	1.136052	0.151744	0.705618	0.057*	
H24B	1.174711	0.259604	0.730684	0.057*	
H24C	1.224817	0.223780	0.642151	0.057*	
S1'	0.76670 (5)	1.04803 (5)	0.53217 (3)	0.02277 (12)	
O1'	1.14505 (15)	0.50110 (16)	0.91923 (11)	0.0362 (4)	
O2'	0.76049 (15)	0.54898 (17)	0.93007 (12)	0.0410 (5)	
H2'	0.782753	0.534895	0.980589	0.078 (12)*	
O3'	0.90933 (13)	0.76045 (13)	0.73711 (9)	0.0234 (3)	
O4'	0.80016 (14)	0.99924 (15)	0.45795 (9)	0.0300 (4)	
O5'	0.75650 (14)	1.17211 (14)	0.52883 (10)	0.0289 (4)	
N1'	0.95957 (17)	0.56602 (18)	0.88347 (12)	0.0302 (4)	
N2'	0.86625 (15)	0.98216 (16)	0.59042 (11)	0.0223 (4)	
C1'	1.0534 (2)	0.4821 (2)	0.90414 (14)	0.0297 (5)	
C2'	1.0214 (2)	0.3675 (2)	0.90349 (14)	0.0306 (5)	
C3'	1.0859 (2)	0.2544 (2)	0.91983 (15)	0.0349 (6)	
H3'	1.162486	0.240626	0.934224	0.042*	
C4'	1.0340 (3)	0.1626 (3)	0.91428 (18)	0.0469 (7)	
H4'	1.075607	0.084206	0.925324	0.056*	
C5'	0.9228 (3)	0.1828 (3)	0.8930 (2)	0.0513 (8)	
H5'	0.889731	0.118235	0.889034	0.062*	
C6'	0.8584 (3)	0.2968 (3)	0.87730 (19)	0.0457 (7)	
H6'	0.781783	0.310803	0.862951	0.055*	
C7'	0.9096 (2)	0.3884 (2)	0.88328 (15)	0.0345 (6)	
C8'	0.8596 (2)	0.5185 (2)	0.87297 (16)	0.0350 (6)	
H8'	0.840457	0.547981	0.815582	0.042*	
C9'	0.9530 (2)	0.6906 (2)	0.87978 (14)	0.0314 (5)	
H9'A	1.003483	0.701044	0.917835	0.038*	
H9'B	0.871471	0.726203	0.899287	0.038*	
C10'	0.98998 (19)	0.7553 (2)	0.79436 (13)	0.0239 (4)	
C11'	1.1054 (2)	0.7027 (2)	0.74680 (14)	0.0260 (5)	
H11'	1.170278	0.652311	0.769535	0.031*	
C12'	1.09712 (19)	0.74141 (19)	0.66761 (14)	0.0251 (5)	
H12'	1.154055	0.724037	0.621769	0.030*	
C13'	0.97764 (19)	0.81916 (19)	0.66568 (13)	0.0221 (4)	
C14'	0.9156 (2)	0.8553 (2)	0.59063 (14)	0.0264 (5)	
H14C	0.971028	0.843925	0.539741	0.032*	
H14D	0.852747	0.809951	0.595442	0.032*	
C15'	0.9874 (2)	0.8886 (2)	0.78895 (13)	0.0255 (5)	
H15C	0.918542	0.924993	0.824380	0.031*	
H15D	1.059722	0.902498	0.804252	0.031*	
C16'	0.97915 (18)	0.93444 (19)	0.69668 (13)	0.0223 (4)	
H16'	1.048276	0.970275	0.668076	0.027*	
C17'	0.86454 (19)	1.01119 (19)	0.67252 (13)	0.0222 (4)	
H17C	0.796071	0.989347	0.711767	0.027*	
H17D	0.864683	1.095695	0.668901	0.027*	
C18'	0.63222 (18)	1.01004 (19)	0.58149 (13)	0.0226 (4)	
C19'	0.6003 (2)	0.9103 (2)	0.56757 (15)	0.0285 (5)	
H19'	0.648619	0.865397	0.529054	0.034*	
C20'	0.4977 (2)	0.8770 (2)	0.61023 (15)	0.0308 (5)	
H20'	0.476141	0.808453	0.601103	0.037*	
C21'	0.4257 (2)	0.9424 (2)	0.66633 (14)	0.0293 (5)	
C22'	0.4569 (2)	1.0438 (2)	0.67779 (14)	0.0283 (5)	
H22'	0.407097	1.090152	0.714863	0.034*	
C23'	0.55990 (19)	1.0784 (2)	0.63577 (13)	0.0248 (4)	
H23'	0.580717	1.147840	0.643989	0.030*	
C24'	0.3143 (2)	0.9045 (3)	0.71372 (16)	0.0400 (6)	
H24D	0.285977	0.947062	0.759935	0.060*	
H24E	0.330163	0.819809	0.734947	0.060*	
H24F	0.254611	0.922022	0.676926	0.060*	

### Atomic displacement parameters (Å^2^)

**Table:**

	*U*^11^	*U*^22^	*U*^33^	*U*^12^	*U*^13^	*U*^23^
S1	0.0286 (3)	0.0229 (3)	0.0220 (3)	−0.0052 (2)	0.0008 (2)	0.0024 (2)
O1	0.0220 (8)	0.0272 (8)	0.0249 (8)	−0.0003 (6)	−0.0020 (6)	0.0004 (6)
O2	0.0233 (8)	0.0326 (9)	0.0245 (8)	−0.0072 (7)	0.0000 (6)	0.0010 (7)
O3	0.0247 (7)	0.0199 (7)	0.0166 (7)	−0.0036 (6)	−0.0020 (6)	0.0007 (5)
O4	0.0367 (10)	0.0216 (8)	0.0409 (10)	−0.0069 (7)	0.0025 (8)	0.0042 (7)
O5	0.0322 (9)	0.0389 (10)	0.0194 (8)	−0.0058 (7)	−0.0017 (7)	0.0023 (7)
N1	0.0260 (9)	0.0207 (9)	0.0175 (8)	−0.0043 (7)	−0.0010 (7)	−0.0003 (7)
N2	0.0269 (9)	0.0236 (9)	0.0188 (9)	−0.0063 (7)	0.0026 (7)	−0.0008 (7)
C1	0.0233 (10)	0.0237 (11)	0.0159 (9)	−0.0033 (8)	−0.0067 (8)	0.0035 (8)
C2	0.0278 (11)	0.0228 (11)	0.0191 (10)	−0.0045 (9)	−0.0076 (8)	0.0015 (8)
C3	0.0327 (12)	0.0272 (12)	0.0253 (11)	−0.0083 (10)	−0.0102 (10)	0.0010 (9)
C4	0.0486 (15)	0.0247 (12)	0.0315 (12)	−0.0112 (11)	−0.0190 (11)	0.0005 (10)
C5	0.0481 (15)	0.0242 (12)	0.0308 (12)	−0.0012 (11)	−0.0153 (11)	−0.0056 (10)
C6	0.0355 (13)	0.0287 (13)	0.0266 (12)	0.0005 (10)	−0.0074 (10)	−0.0043 (9)
C7	0.0291 (11)	0.0249 (11)	0.0170 (10)	−0.0030 (9)	−0.0047 (8)	−0.0013 (8)
C8	0.0254 (11)	0.0266 (11)	0.0179 (10)	−0.0025 (9)	0.0002 (8)	−0.0013 (8)
C9	0.0294 (11)	0.0218 (11)	0.0191 (10)	−0.0056 (9)	−0.0030 (9)	−0.0012 (8)
C10	0.0252 (10)	0.0208 (10)	0.0176 (9)	−0.0045 (8)	−0.0009 (8)	−0.0015 (8)
C11	0.0244 (11)	0.0292 (12)	0.0262 (11)	−0.0067 (9)	−0.0030 (9)	0.0018 (9)
C12	0.0265 (11)	0.0341 (13)	0.0247 (11)	−0.0072 (10)	−0.0071 (9)	0.0027 (9)
C13	0.0280 (11)	0.0201 (10)	0.0181 (10)	−0.0034 (8)	−0.0033 (8)	0.0018 (8)
C14	0.0342 (12)	0.0264 (12)	0.0195 (10)	−0.0110 (9)	−0.0011 (9)	−0.0015 (8)
C15	0.0360 (13)	0.0226 (11)	0.0203 (10)	−0.0070 (9)	0.0042 (9)	−0.0042 (8)
C16	0.0315 (12)	0.0203 (11)	0.0219 (10)	−0.0025 (9)	0.0005 (9)	0.0010 (8)
C17	0.0348 (12)	0.0205 (11)	0.0231 (11)	−0.0080 (9)	0.0015 (9)	−0.0016 (8)
C18	0.0260 (11)	0.0236 (11)	0.0203 (10)	−0.0068 (9)	0.0008 (8)	−0.0027 (8)
C19	0.0336 (12)	0.0250 (12)	0.0320 (12)	−0.0099 (10)	−0.0008 (10)	−0.0082 (9)
C20	0.0343 (13)	0.0346 (13)	0.0267 (11)	−0.0146 (10)	−0.0075 (10)	−0.0081 (10)
C21	0.0258 (11)	0.0324 (13)	0.0220 (11)	−0.0098 (9)	−0.0007 (9)	−0.0021 (9)
C22	0.0282 (11)	0.0237 (11)	0.0252 (11)	−0.0052 (9)	−0.0006 (9)	−0.0063 (9)
C23	0.0261 (11)	0.0269 (12)	0.0236 (11)	−0.0071 (9)	−0.0045 (9)	−0.0074 (9)
C24	0.0322 (13)	0.0454 (16)	0.0372 (14)	−0.0042 (11)	−0.0143 (11)	−0.0033 (12)
S1'	0.0233 (3)	0.0244 (3)	0.0192 (2)	−0.0058 (2)	−0.0031 (2)	0.0010 (2)
O1'	0.0288 (9)	0.0474 (11)	0.0299 (9)	−0.0071 (8)	−0.0058 (7)	0.0000 (8)
O2'	0.0231 (9)	0.0504 (12)	0.0405 (11)	−0.0009 (8)	−0.0048 (8)	0.0061 (8)
O3'	0.0216 (7)	0.0276 (8)	0.0200 (7)	−0.0079 (6)	−0.0011 (6)	−0.0002 (6)
O4'	0.0309 (9)	0.0383 (10)	0.0192 (8)	−0.0050 (7)	−0.0038 (7)	−0.0029 (7)
O5'	0.0312 (9)	0.0232 (8)	0.0311 (9)	−0.0088 (7)	−0.0046 (7)	0.0023 (6)
N1'	0.0264 (10)	0.0315 (11)	0.0270 (10)	−0.0065 (8)	−0.0029 (8)	0.0076 (8)
N2'	0.0214 (9)	0.0258 (10)	0.0188 (8)	−0.0025 (7)	−0.0037 (7)	−0.0031 (7)
C1'	0.0250 (11)	0.0389 (14)	0.0186 (10)	−0.0036 (10)	0.0003 (9)	0.0041 (9)
C2'	0.0254 (11)	0.0362 (14)	0.0245 (11)	−0.0044 (10)	0.0001 (9)	0.0031 (10)
C3'	0.0320 (13)	0.0372 (14)	0.0260 (12)	−0.0002 (11)	0.0043 (10)	0.0019 (10)
C4'	0.0482 (17)	0.0344 (15)	0.0467 (17)	−0.0011 (13)	0.0068 (14)	−0.0007 (12)
C5'	0.0491 (18)	0.0444 (18)	0.060 (2)	−0.0168 (14)	0.0031 (15)	−0.0084 (15)
C6'	0.0356 (15)	0.0487 (18)	0.0520 (17)	−0.0157 (13)	−0.0054 (13)	0.0007 (14)
C7'	0.0292 (12)	0.0395 (15)	0.0292 (12)	−0.0070 (11)	−0.0008 (10)	0.0046 (10)
C8'	0.0248 (12)	0.0422 (15)	0.0323 (13)	−0.0055 (10)	−0.0053 (10)	0.0067 (11)
C9'	0.0330 (13)	0.0342 (13)	0.0224 (11)	−0.0049 (10)	−0.0003 (10)	0.0009 (9)
C10'	0.0222 (10)	0.0295 (12)	0.0185 (10)	−0.0059 (9)	−0.0027 (8)	0.0000 (8)
C11'	0.0236 (11)	0.0256 (11)	0.0267 (11)	−0.0035 (9)	−0.0027 (9)	−0.0017 (9)
C12'	0.0227 (11)	0.0254 (11)	0.0254 (11)	−0.0030 (9)	−0.0006 (9)	−0.0044 (9)
C13'	0.0235 (10)	0.0245 (11)	0.0178 (10)	−0.0066 (8)	−0.0005 (8)	−0.0025 (8)
C14'	0.0311 (12)	0.0262 (12)	0.0228 (11)	−0.0009 (9)	−0.0070 (9)	−0.0080 (9)
C15'	0.0261 (11)	0.0321 (12)	0.0198 (10)	−0.0067 (9)	−0.0049 (9)	−0.0051 (9)
C16'	0.0214 (10)	0.0252 (11)	0.0209 (10)	−0.0074 (8)	−0.0025 (8)	−0.0026 (8)
C17'	0.0239 (10)	0.0227 (11)	0.0200 (10)	−0.0057 (8)	−0.0015 (8)	−0.0041 (8)
C18'	0.0201 (10)	0.0235 (11)	0.0226 (10)	−0.0039 (8)	−0.0058 (8)	0.0017 (8)
C19'	0.0285 (12)	0.0235 (11)	0.0342 (12)	−0.0032 (9)	−0.0086 (10)	−0.0053 (9)
C20'	0.0305 (12)	0.0259 (12)	0.0389 (14)	−0.0104 (10)	−0.0138 (10)	0.0009 (10)
C21'	0.0239 (11)	0.0348 (13)	0.0276 (12)	−0.0092 (9)	−0.0134 (9)	0.0103 (10)
C22'	0.0234 (11)	0.0358 (13)	0.0236 (11)	−0.0022 (9)	−0.0055 (9)	−0.0021 (9)
C23'	0.0248 (11)	0.0257 (11)	0.0239 (11)	−0.0057 (9)	−0.0051 (9)	−0.0021 (8)
C24'	0.0281 (13)	0.0547 (17)	0.0358 (14)	−0.0165 (12)	−0.0121 (11)	0.0109 (12)

### Geometric parameters (Å, º)

**Table:**

S1—O4	1.4332 (17)	S1'—O4'	1.4337 (17)
S1—O5	1.4337 (17)	S1'—O5'	1.4378 (17)
S1—N2	1.6368 (19)	S1'—N2'	1.6263 (18)
S1—C18	1.758 (2)	S1'—C18'	1.758 (2)
O1—C1	1.240 (3)	O1'—C1'	1.231 (3)
O2—C8	1.407 (3)	O2'—C8'	1.404 (3)
O2—H2	0.9160	O2'—H2'	0.9026
O3—C10	1.449 (2)	O3'—C13'	1.452 (2)
O3—C13	1.456 (2)	O3'—C10'	1.453 (3)
N1—C1	1.356 (3)	N1'—C1'	1.363 (3)
N1—C9	1.452 (3)	N1'—C9'	1.449 (3)
N1—C8	1.477 (3)	N1'—C8'	1.466 (3)
N2—C17	1.481 (3)	N2'—C17'	1.480 (3)
N2—C14	1.487 (3)	N2'—C14'	1.488 (3)
C1—C2	1.474 (3)	C1'—C2'	1.488 (4)
C2—C7	1.387 (3)	C2'—C7'	1.382 (3)
C2—C3	1.390 (3)	C2'—C3'	1.389 (3)
C3—C4	1.394 (3)	C3'—C4'	1.385 (4)
C3—H3	0.9500	C3'—H3'	0.9500
C4—C5	1.391 (4)	C4'—C5'	1.381 (4)
C4—H4	0.9500	C4'—H4'	0.9500
C5—C6	1.393 (4)	C5'—C6'	1.396 (4)
C5—H5	0.9500	C5'—H5'	0.9500
C6—C7	1.383 (3)	C6'—C7'	1.379 (4)
C6—H6	0.9500	C6'—H6'	0.9500
C7—C8	1.511 (3)	C7'—C8'	1.511 (4)
C8—H8	1.0000	C8'—H8'	1.0000
C9—C10	1.511 (3)	C9'—C10'	1.510 (3)
C9—H9A	0.9900	C9'—H9'A	0.9900
C9—H9B	0.9900	C9'—H9'B	0.9900
C10—C11	1.516 (3)	C10'—C11'	1.526 (3)
C10—C15	1.563 (3)	C10'—C15'	1.557 (3)
C11—C12	1.325 (3)	C11'—C12'	1.323 (3)
C11—H11	0.9500	C11'—H11'	0.9500
C12—C13	1.502 (3)	C12'—C13'	1.512 (3)
C12—H12	0.9500	C12'—H12'	0.9500
C13—C14	1.509 (3)	C13'—C14'	1.507 (3)
C13—C16	1.565 (3)	C13'—C16'	1.563 (3)
C14—H14A	0.9900	C14'—H14C	0.9900
C14—H14B	0.9900	C14'—H14D	0.9900
C15—C16	1.546 (3)	C15'—C16'	1.540 (3)
C15—H15A	0.9900	C15'—H15C	0.9900
C15—H15B	0.9900	C15'—H15D	0.9900
C16—C17	1.525 (3)	C16'—C17'	1.529 (3)
C16—H16	1.0000	C16'—H16'	1.0000
C17—H17A	0.9900	C17'—H17C	0.9900
C17—H17B	0.9900	C17'—H17D	0.9900
C18—C19	1.388 (3)	C18'—C19'	1.389 (3)
C18—C23	1.391 (3)	C18'—C23'	1.392 (3)
C19—C20	1.387 (3)	C19'—C20'	1.383 (3)
C19—H19	0.9500	C19'—H19'	0.9500
C20—C21	1.392 (3)	C20'—C21'	1.391 (4)
C20—H20	0.9500	C20'—H20'	0.9500
C21—C22	1.389 (3)	C21'—C22'	1.388 (3)
C21—C24	1.504 (3)	C21'—C24'	1.515 (3)
C22—C23	1.387 (3)	C22'—C23'	1.389 (3)
C22—H22	0.9500	C22'—H22'	0.9500
C23—H23	0.9500	C23'—H23'	0.9500
C24—H24A	0.9800	C24'—H24D	0.9800
C24—H24B	0.9800	C24'—H24E	0.9800
C24—H24C	0.9800	C24'—H24F	0.9800
			
O4—S1—O5	120.45 (10)	O4'—S1'—O5'	120.20 (10)
O4—S1—N2	106.71 (10)	O4'—S1'—N2'	105.39 (10)
O5—S1—N2	105.53 (10)	O5'—S1'—N2'	106.73 (10)
O4—S1—C18	107.32 (11)	O4'—S1'—C18'	108.51 (10)
O5—S1—C18	109.11 (10)	O5'—S1'—C18'	107.32 (10)
N2—S1—C18	107.01 (10)	N2'—S1'—C18'	108.20 (10)
C8—O2—H2	108.3	C8'—O2'—H2'	108.3
C10—O3—C13	95.72 (15)	C13'—O3'—C10'	95.37 (15)
C1—N1—C9	123.97 (18)	C1'—N1'—C9'	123.9 (2)
C1—N1—C8	113.04 (18)	C1'—N1'—C8'	113.8 (2)
C9—N1—C8	122.98 (17)	C9'—N1'—C8'	122.0 (2)
C17—N2—C14	109.96 (16)	C17'—N2'—C14'	110.14 (16)
C17—N2—S1	119.69 (15)	C17'—N2'—S1'	121.96 (14)
C14—N2—S1	118.46 (14)	C14'—N2'—S1'	119.12 (14)
O1—C1—N1	125.2 (2)	O1'—C1'—N1'	125.3 (2)
O1—C1—C2	127.7 (2)	O1'—C1'—C2'	128.7 (2)
N1—C1—C2	107.04 (18)	N1'—C1'—C2'	106.0 (2)
C7—C2—C3	121.9 (2)	C7'—C2'—C3'	121.8 (2)
C7—C2—C1	108.71 (19)	C7'—C2'—C1'	108.7 (2)
C3—C2—C1	129.4 (2)	C3'—C2'—C1'	129.6 (2)
C2—C3—C4	117.1 (2)	C4'—C3'—C2'	117.2 (3)
C2—C3—H3	121.5	C4'—C3'—H3'	121.4
C4—C3—H3	121.5	C2'—C3'—H3'	121.4
C5—C4—C3	120.9 (2)	C5'—C4'—C3'	121.3 (3)
C5—C4—H4	119.5	C5'—C4'—H4'	119.3
C3—C4—H4	119.5	C3'—C4'—H4'	119.3
C4—C5—C6	121.5 (2)	C4'—C5'—C6'	121.0 (3)
C4—C5—H5	119.2	C4'—C5'—H5'	119.5
C6—C5—H5	119.2	C6'—C5'—H5'	119.5
C7—C6—C5	117.5 (2)	C7'—C6'—C5'	117.8 (3)
C7—C6—H6	121.3	C7'—C6'—H6'	121.1
C5—C6—H6	121.3	C5'—C6'—H6'	121.1
C6—C7—C2	121.1 (2)	C6'—C7'—C2'	120.8 (3)
C6—C7—C8	129.4 (2)	C6'—C7'—C8'	129.4 (2)
C2—C7—C8	109.48 (19)	C2'—C7'—C8'	109.8 (2)
O2—C8—N1	112.16 (17)	O2'—C8'—N1'	112.0 (2)
O2—C8—C7	114.70 (18)	O2'—C8'—C7'	114.4 (2)
N1—C8—C7	101.68 (17)	N1'—C8'—C7'	101.48 (19)
O2—C8—H8	109.3	O2'—C8'—H8'	109.6
N1—C8—H8	109.3	N1'—C8'—H8'	109.6
C7—C8—H8	109.3	C7'—C8'—H8'	109.6
N1—C9—C10	113.26 (17)	N1'—C9'—C10'	113.6 (2)
N1—C9—H9A	108.9	N1'—C9'—H9'A	108.9
C10—C9—H9A	108.9	C10'—C9'—H9'A	108.9
N1—C9—H9B	108.9	N1'—C9'—H9'B	108.9
C10—C9—H9B	108.9	C10'—C9'—H9'B	108.9
H9A—C9—H9B	107.7	H9'A—C9'—H9'B	107.7
O3—C10—C9	111.77 (17)	O3'—C10'—C9'	112.69 (18)
O3—C10—C11	101.29 (16)	O3'—C10'—C11'	100.79 (17)
C9—C10—C11	118.54 (18)	C9'—C10'—C11'	117.59 (19)
O3—C10—C15	100.25 (15)	O3'—C10'—C15'	100.44 (16)
C9—C10—C15	115.28 (18)	C9'—C10'—C15'	114.75 (19)
C11—C10—C15	107.33 (18)	C11'—C10'—C15'	108.40 (18)
C12—C11—C10	106.5 (2)	C12'—C11'—C10'	106.32 (19)
C12—C11—H11	126.7	C12'—C11'—H11'	126.8
C10—C11—H11	126.7	C10'—C11'—H11'	126.8
C11—C12—C13	105.4 (2)	C11'—C12'—C13'	105.22 (19)
C11—C12—H12	127.3	C11'—C12'—H12'	127.4
C13—C12—H12	127.3	C13'—C12'—H12'	127.4
O3—C13—C12	102.11 (16)	O3'—C13'—C14'	113.05 (18)
O3—C13—C14	111.92 (18)	O3'—C13'—C12'	101.73 (16)
C12—C13—C14	125.30 (19)	C14'—C13'—C12'	124.03 (19)
O3—C13—C16	100.04 (16)	O3'—C13'—C16'	99.92 (16)
C12—C13—C16	107.30 (18)	C14'—C13'—C16'	106.76 (17)
C14—C13—C16	107.29 (17)	C12'—C13'—C16'	108.74 (17)
N2—C14—C13	103.49 (17)	N2'—C14'—C13'	103.46 (17)
N2—C14—H14A	111.1	N2'—C14'—H14C	111.1
C13—C14—H14A	111.1	C13'—C14'—H14C	111.1
N2—C14—H14B	111.1	N2'—C14'—H14D	111.1
C13—C14—H14B	111.1	C13'—C14'—H14D	111.1
H14A—C14—H14B	109.0	H14C—C14'—H14D	109.0
C16—C15—C10	100.84 (17)	C16'—C15'—C10'	100.90 (17)
C16—C15—H15A	111.6	C16'—C15'—H15C	111.6
C10—C15—H15A	111.6	C10'—C15'—H15C	111.6
C16—C15—H15B	111.6	C16'—C15'—H15D	111.6
C10—C15—H15B	111.6	C10'—C15'—H15D	111.6
H15A—C15—H15B	109.4	H15C—C15'—H15D	109.4
C17—C16—C15	119.1 (2)	C17'—C16'—C15'	118.39 (18)
C17—C16—C13	101.38 (17)	C17'—C16'—C13'	101.21 (16)
C15—C16—C13	101.55 (17)	C15'—C16'—C13'	101.62 (17)
C17—C16—H16	111.2	C17'—C16'—H16'	111.5
C15—C16—H16	111.2	C15'—C16'—H16'	111.5
C13—C16—H16	111.2	C13'—C16'—H16'	111.5
N2—C17—C16	101.58 (18)	N2'—C17'—C16'	100.82 (16)
N2—C17—H17A	111.5	N2'—C17'—H17C	111.6
C16—C17—H17A	111.5	C16'—C17'—H17C	111.6
N2—C17—H17B	111.5	N2'—C17'—H17D	111.6
C16—C17—H17B	111.5	C16'—C17'—H17D	111.6
H17A—C17—H17B	109.3	H17C—C17'—H17D	109.4
C19—C18—C23	120.8 (2)	C19'—C18'—C23'	120.5 (2)
C19—C18—S1	120.11 (18)	C19'—C18'—S1'	119.70 (17)
C23—C18—S1	119.01 (17)	C23'—C18'—S1'	119.76 (17)
C20—C19—C18	119.2 (2)	C20'—C19'—C18'	119.4 (2)
C20—C19—H19	120.4	C20'—C19'—H19'	120.3
C18—C19—H19	120.4	C18'—C19'—H19'	120.3
C19—C20—C21	121.0 (2)	C19'—C20'—C21'	120.9 (2)
C19—C20—H20	119.5	C19'—C20'—H20'	119.5
C21—C20—H20	119.5	C21'—C20'—H20'	119.5
C22—C21—C20	118.8 (2)	C22'—C21'—C20'	119.0 (2)
C22—C21—C24	119.4 (2)	C22'—C21'—C24'	120.3 (2)
C20—C21—C24	121.7 (2)	C20'—C21'—C24'	120.7 (2)
C23—C22—C21	121.2 (2)	C21'—C22'—C23'	120.9 (2)
C23—C22—H22	119.4	C21'—C22'—H22'	119.6
C21—C22—H22	119.4	C23'—C22'—H22'	119.6
C22—C23—C18	119.0 (2)	C22'—C23'—C18'	119.2 (2)
C22—C23—H23	120.5	C22'—C23'—H23'	120.4
C18—C23—H23	120.5	C18'—C23'—H23'	120.4
C21—C24—H24A	109.5	C21'—C24'—H24D	109.5
C21—C24—H24B	109.5	C21'—C24'—H24E	109.5
H24A—C24—H24B	109.5	H24D—C24'—H24E	109.5
C21—C24—H24C	109.5	C21'—C24'—H24F	109.5
H24A—C24—H24C	109.5	H24D—C24'—H24F	109.5
H24B—C24—H24C	109.5	H24E—C24'—H24F	109.5

### Hydrogen-bond geometry (Å, º)

**Table:**

*D*—H···*A*	*D*—H	H···*A*	*D*···*A*	*D*—H···*A*
C8—H8···O3	1.00	2.63	3.206 (3)	117
C12—H12···O5^i^	0.95	2.47	3.333 (3)	151
C14—H14*A*···O5′^ii^	0.99	2.62	3.506 (3)	149
C23—H23···O5′^ii^	0.95	2.41	3.195 (3)	139
C14′—H14*D*···O4	0.99	2.58	3.482 (3)	151
C15′—H15*C*···O2^iii^	0.99	2.62	3.585 (3)	165
C16′—H16′···O4′^iv^	1.00	2.53	3.422 (3)	149
C19′—H19′···O4	0.95	2.34	3.078 (3)	134
O2—H2···O1^v^	0.92	1.85	2.756 (2)	172
O2′—H2′···O1′^vi^	0.90	1.95	2.840 (3)	171

Symmetry codes: (i) −*x*+1, −*y*+1, −*z*+1; (ii) *x*, *y*−1, *z*; (iii) *x*, *y*+1, *z*; (iv) −*x*+2, −*y*+2, −*z*+1; (v) −*x*+1, −*y*, −*z*+2; (vi) −*x*+2, −*y*+1, −*z*+2.
